# Coherent suppression of backscattering in optical microresonators

**DOI:** 10.1038/s41377-020-00440-2

**Published:** 2020-12-23

**Authors:** Andreas Ø. Svela, Jonathan M. Silver, Leonardo Del Bino, Shuangyou Zhang, Michael T. M. Woodley, Michael R. Vanner, Pascal Del’Haye

**Affiliations:** 1 National Physical Laboratory, Teddington, TW11 0LW UK; 2 Blackett Laboratory, Imperial College London, London, SW7 2AZ UK; 3 Clarendon Laboratory, University of Oxford, Oxford, OX1 3PU UK; 4 City University of London, London, EC1V 0HB UK; 5 Max Planck Institute for the Science of Light, Staudtstaße 2, 91058 Erlangen, Germany; 6 Heriot-Watt University, Edinburgh, EH14 4AS UK; 7 Friedrich Alexander University Erlangen-Nuremberg, 91058 Erlangen, Germany

**Keywords:** Optical metrology, Sub-wavelength optics, Optical sensors, Microresonators

## Abstract

As light propagates along a waveguide, a fraction of the field can be reflected by Rayleigh scatterers. In high-quality-factor whispering-gallery-mode microresonators, this intrinsic backscattering is primarily caused by either surface or bulk material imperfections. For several types of microresonator-based experiments and applications, minimal backscattering in the cavity is of critical importance, and thus, the ability to suppress backscattering is essential. We demonstrate that the introduction of an additional scatterer into the near field of a high-quality-factor microresonator can coherently suppress the amount of backscattering in the microresonator by more than 30 dB. The method relies on controlling the scatterer position such that the intrinsic and scatterer-induced backpropagating fields destructively interfere. This technique is useful in microresonator applications where backscattering is currently limiting the performance of devices, such as ring-laser gyroscopes and dual frequency combs, which both suffer from injection locking. Moreover, these findings are of interest for integrated photonic circuits in which back reflections could negatively impact the stability of laser sources or other components.

## Optics: suppressing backscattering improves performance of microresonators

A technique that suppresses backscattering in optical whispering-gallery-mode (WGM) microresonators significantly improves their performance, opening the door for their use in photonic devices for various applications. Imperfections in the microresonator surface or bulk material can cause backscattering of a portion of the light into the counterpropagating WGM, limiting their performance. A team of researchers led by Pascal Del’Haye from the Max Planck Institute for the Science of Light in Germany have now demonstrated a technique that can suppress backscatter by more than 30 decibels. By introducing an additional scatterer in the near-field of a high-quality-factor WGM microresonator, the researchers could control the scatterer’s position so that the intrinsic and scatter-induced backpropagating fields interfere destructively. The technique could be used for photonic devices in which minimal backscattering is essential, such as laser gyroscopes and dual-frequency combs.

## Introduction

Optical whispering-gallery-mode (WGM) microresonators are widely used in photonics for a range of applications, including sensing and metrology^1–6^, optomechanics^7–9^, quantum optics^10,11^, and classical and quantum information processing^12–14^. Microresonators can adopt a range of different geometries, but for all of them, imperfections in the resonator surface or bulk material can cause scattering of some portion of the light into the counter-propagating whispering-gallery mode^15–18^.

Backscattered light in microresonators can limit the application performance, for example, causing unwanted injection locking in laser gyroscopes operating at low rotational speeds^19–21^ or in dual frequency combs^22,23^. Backscattering also reduces the nonlinear enhancement and contributes to back reflections in devices relying on symmetry breaking of counter-propagating fields^24^ for sensing^25^, optical computing^26,27^ or isolator^28^ applications. Furthermore, control over backscattering permits tuning of the standing wave pattern to maximise coupling by moving an anti-node of the standing wave along the resonator perimeter, which is beneficial for systems relying on evanescent coupling, such as evanescent optomechanics^8^, or biomedical near-field sensors^29^. In addition, telecom applications^14,30^ can benefit from lower backscattering levels.

The imperfections causing backscattering in microresonators are typically distributed around the cavity but can be approximated as a single scatterer with specific amplitude and phase, as the coherence length of the circulating field is much longer than the cavity round-trip length^31^. The reflected field is resonant in the cavity and therefore builds up in the counter-propagating direction^32,33^. This coupling of the two travelling-wave modes generally results in non-orthogonal chiral eigenmodes composed of unequal superpositions of the two travelling-wave modes^34^. These superposition modes typically have different frequencies and losses. For high levels of backscattering, the mode splitting may be spectrally resolvable, i.e., detected as two separate resonances^16,18^. Previous experimental work has focused on controlling and changing the backscattering in such systems with intrinsically high backscattering rates, tuning the mode splitting with a near-field scatterer^35,36^ or inducing chirality for light flow control^37,38^, without investigating backscattering suppression. The backscattering problem is now attracting interest in the community, and recently, an optomechanical method to reduce backscattering was demonstrated^39^, showing suppression from resolved to unresolved mode splitting.

**Fig. 1:**
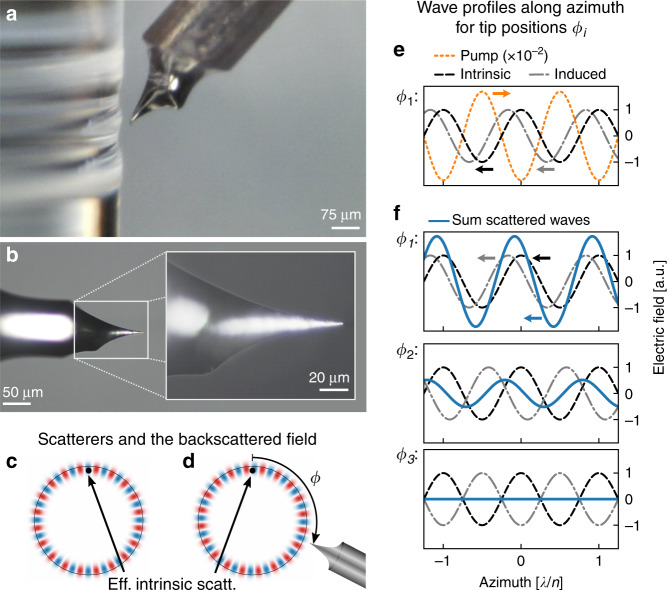
Principle of backscattering control. **a**–**b** Micrographs of the microresonator and tungsten tip. **c** Illustration of the real part of the backscattered field (cavity size and wavelength not to scale); the scatterer is represented by one effective scatterer here (black dot). **d** Introducing a second scatterer at an azimuthal angular distance *ϕ* from the effective scatterer, the backscattered field changes depending on *ϕ*. **e** Illustration of wave profiles along the azimuth showing the counter-clockwise-propagating backscattered waves due to the clockwise-propagating pump field. The backscattered field amplitudes are small compared to the pump, as only a small fraction of the light is backscattered. The tip is *critically coupled* (equal amplitudes for intrinsic and induced backscattered waves). **f** Backscattered waves and their sum under critical coupling (equal amplitudes) for different azimuthal positions *ϕ*_*i*_ of the tip, corresponding to phase offsets between the effective intrinsic scatterer and the induced scatterer (2 *m* + *q*) *π* for integer *m* and *q* = 1*/*3, 5*/*6 and 1, showing both constructive and destructive interference

Here, we show an unprecedented 34 dB suppression of the backscattered light from a WGM resonator, limited by the photodetector noise. This is achieved by manipulating the position of a sub-wavelength-size scatterer within the near field of the optical mode (Fig. 1a), coherently controlling the effective backscattering. We demonstrate the effect in two silica rod microresonators with intrinsically low backscattering, meaning neither one shows resolved frequency mode splitting (resonator diameters *d* = 2.7 mm, 1.7 mm and *Q* $\simeq$ 2 × 10^8^, 1.1 × 10^9^).

In our setup, a sub-wavelength tungsten tip (Fig. 1b–d) controls the backscattering by coherently scattering additional light from the pumped optical mode into the counter-propagating mode (Fig. 1e), leading to interference between the intrinsic backscattering and that caused by the metal tip^34^. With sufficient induced backscattering and an appropriate phase offset between the intrinsic and induced backscattered waves, the net backscattering can be made to vanish. As the radial position of the tip controls the amount of induced backscattering and the azimuthal position governs the phase offset between the intrinsic and induced backscattered waves, the tip position can coherently control the net backscattered field (Fig. 1f). When the tip induces backscattering of equal magnitude to the intrinsic backscattering, we call this *critical tip coupling*. The tip also scatters to free-space modes; however, for a small tip diameter, the reduction in the Q-factor is small. This technique enables full control of the amplitude and phase of the effective backscattering in a microresonator, and in this experiment, we show that it can be reduced by orders of magnitude beyond the unresolved frequency splitting level.

## Results

### Response of the resonator to a perturbation in the near field

**Fig. 2:**
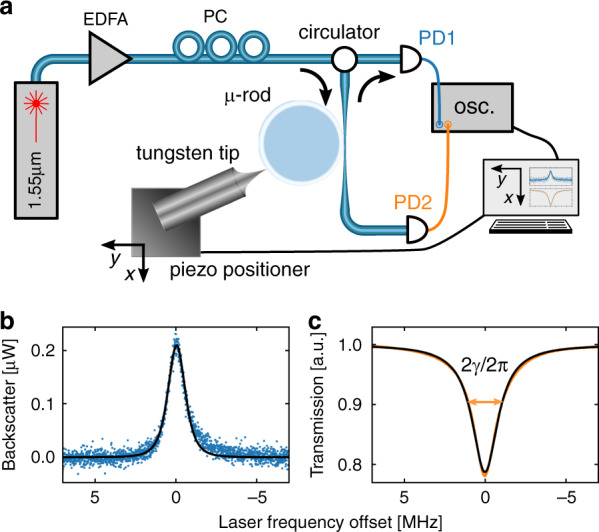
Experimental setup and example measurements. **a** Optical circuitry, consisting of a fibre-coupled external cavity diode laser (1.55 µm) amplified by an erbium-doped fibre amplifier (EDFA), a polarisation controller (PC) to optimise the coupling to the desired resonator mode, a circulator to separate the propagation directions in the fibre, and photodetectors to monitor the backscattering (PD1) and transmission of the microresonator (PD2). The tungsten tip near-field probe was fixed to a piezo positioner. Example backscattering **b** and transmission **c** spectra with fitted Lorentzians (black) for one position of the near-field probe, showing the transmission linewidth 2*γ*

We studied the backscattering amplitude and resonance linewidth as functions of the distance of the tungsten tip from the resonator surface *r* and its azimuthal position *ϕ* while keeping the tip in the resonator plane (*xy* plane in Fig. 2a). The microrod resonator was pumped with a tuneable, 1.55 µm continuous-wave laser using a tapered optical fibre^40^ to couple light into the cavity. The 20 mW input optical power was scanned downwards in frequency to obtain spectral data of the resonance. To avoid thermal broadening^41^ of the resonance, the laser frequency was scanned at a rate of 450 GHz s^−1^. The nonlinear Kerr effect is faster than the scanning rate and thus causes some broadening; however, with low input power, this broadening is small. A circulator allowed the backscattered light from the cavity to be monitored with a photodetector, along with the cavity transmission (see Fig. 2a). The field-perturbing probe was fixed to a computer-controlled piezoelectric positioner for three-axis control of the tip position.

**Fig. 3:**
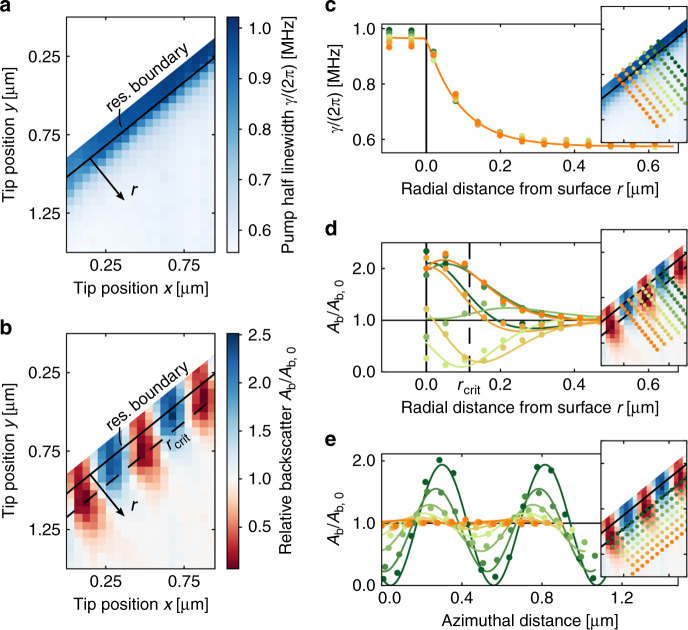
Resonator response to the tungsten tip in the near field. **a** Total linewidth (and equivalently Q-factor) and **b** backscattering amplitude for tip positions in the resonator plane, normalised by the intrinsic backscattering amplitude *A*_b,0_. The black lines (*r* = 0) indicate the fitted resonator surface–measurements shown as *r* *<* 0 were obtained while the tip was touching the resonator surface, resulting in the tip sliding along the surface. Maximum backscattering suppression is found along the dashed line (*critical tip coupling*); for *r* *<* *r*_crit_, the tip is *over-coupled*, reducing the suppression. **c**–**e** Fits (lines) and interpolated data (circles) along cross sections through the experimental data in panels a and b. The insets show where the cross sections were taken. **c** Radial tip position dependence of the linewidth and **d** backscattering. **e** Tip azimuthal position dependence of the backscattering

The tip position was raster scanned with a step size of 50 nm over a grid of (*x, y*) positions in the resonator plane. At each position, transmission and backscattering spectra were simultaneously recorded. The spectra were subsequently fitted with Lorentzian functions (see “Materials and methods” section), as shown for the examples in Fig. 2b, c, to extract the backscattering Lorentzian amplitude *A*_b_ and the pump resonance half-linewidth *γ*, respectively. The resulting data grids for the resonator with *Q* $\simeq$ 2 × 10^8^ are shown in Fig. 3a, b, where each pixel corresponds to a position on the measurement grid. For positions corresponding to *r* < 0, the tip was touching the resonator and sliding along its surface due to the force applied by the piezo positioner.

We performed numerical fitting of the linewidth and amplitude data vs. the two spatial coordinates to determine the position of the resonator surface and radial distance dependences and estimate the periodicity in the fringe pattern and suppression achieved for the backscattering amplitude.

### The near-field decay and resonator boundary

Assuming a linear coupling between the evanescent near field and the tip, we expect the radial dependence of the linewidth to have the same functional dependence as the energy density in the near field. The evanescent electric field from a waveguide decays exponentially with the perpendicular distance from the surface—i.e., for a WGM resonator, with the radial distance from the surface, *r*. The evanescent field can be expressed as $E_{{\mathrm{ev}}}\left( r \right) = E_{{\mathrm{surf}}}{\mathrm{exp}}( - \alpha r)$, where *E*_surf_ is the field strength at the surface, and the decay length is

1 $$\begin{array}{*{20}{c}} {\alpha ^{ - 1} = \frac{\lambda }{{2\pi \sqrt {n^2 - 1} }}} \end{array}$$

for a field of vacuum wavelength *λ* in a waveguide of refractive index *n* surrounded by air^42^. With the evanescent field energy density proportional to $\left| {E_{{\mathrm{ev}}}\left( r \right)} \right|^2 \propto {\mathrm{exp}}( - 2\alpha r)$, we expect the tip-induced loss and backscattering amplitude to also be proportional to this quantity.

However, the prefactors corresponding to coupling back to the resonator clockwise and counter-clockwise directions, coupling to free-space modes, and absorption by the tip are dependent on the size, geometry, and material of the scatterer, as well as on the polarisation of the mode. As propagating modes of different orders than the mode in question have different resonance frequencies, we do not expect light to couple to other WGM modes. Note that in addition to coupling into free space modes, the tip could also induce losses via coupling into non-guided modes within the fused silica. It has previously been shown that for a silica sub-wavelength tip, it is the tip size relative to the mode volume that determines the amount of induced losses^35^, and furthermore, the tip can cause mode splitting with no change in the quality factor compared to when the tip is not present^34,43^.

The linewidth data were fitted with a piecewise function comprising an exponential decay from the resonator surface and a linear plateau for the tip positions *r* < 0 where the tip is touching the resonator surface (detailed fitting function given in “Materials and methods” section). The fitted interface between the plateau and the exponential decay determines the resonator surface *r* = 0, shown as black lines in Fig. 3a, b. Figure 3c shows cross sections of interpolated values (circles) and the fit (solid) to the linewidth data along the radial direction. The fit gives an exponential decay length for the linewidth of (2*α*_*γ*_)^−1^ = 92 nm, compared to the calculated near-field power decay length of (2*α*)^−1^ = 119 nm using Eq. (1) for silica, *n* = 1.44 at *λ* = 1553 nm. The steeper decay coefficient in the experimental data compared to the calculated value can be explained by the tip geometry; as the tip approaches the surface, the effective scattering cross section/polarisability increases, leading to an increasingly larger broadening.

### Backscattering suppression analysis

The backscattering data in Fig. 3b show maxima and minima when the tip is some distance from the resonator rather than at the surface owing to *over-coupling* of the tip—i.e., the induced backscattering is larger than the intrinsic backscattering. The radial distance at which the tip minimises the backscattering, *r*_crit_, is indicated. The data *r* ≥ 0 are fitted with a function that is effectively an exponential decay of coefficient 2*α*_b_ multiplied by a fringe pattern (see “Materials and methods” section). The fringe pattern arises due to the relative phase change between the intrinsic and induced backscattering as the tip is translated along the azimuthal direction. The expected periodicity of the fringe pattern can be estimated by *λ*/2*n* = 539 nm for silica at our pump wavelength (see Supplementary information). Figure 3d, e show cross sections of interpolated values (circles) and the fit (solid) along the radial and azimuthal directions, respectively, for the backscattering data. The fit gives a fringe period of 515 nm and a decay coefficient for the backscattering of (2*α*_b_)^−1^ = 99 nm, similar to the linewidth decay length. The current setup is stable enough for the fringe pattern position to remain in place for measurements of at least 45 min.

### Backscattering suppression in resonators with higher Q-factors

**Fig. 4:**
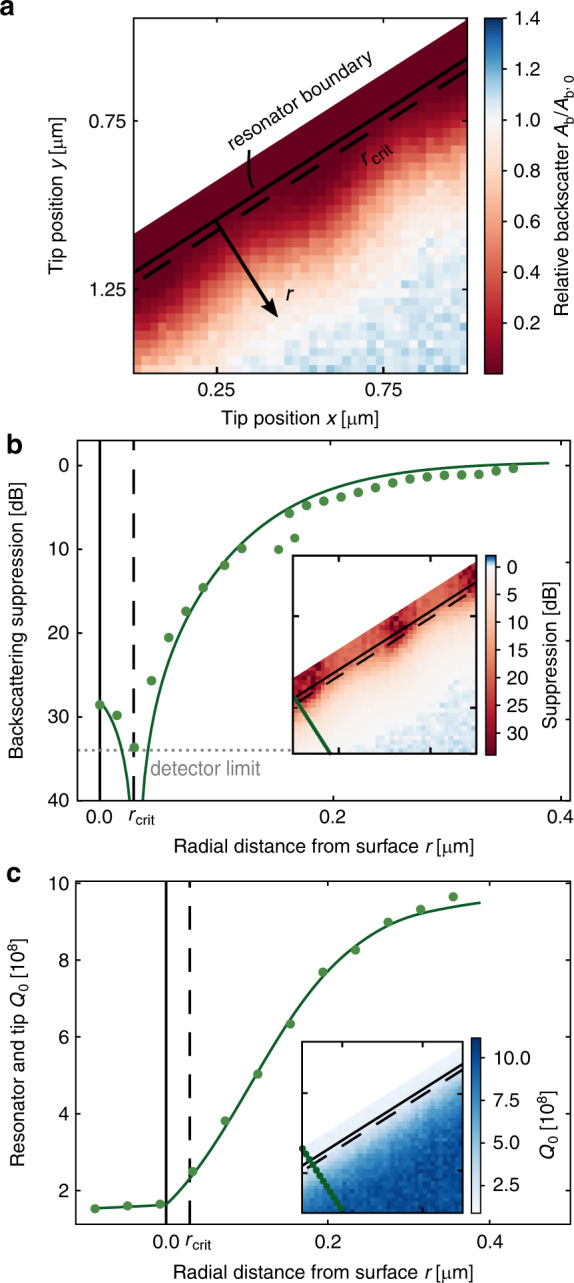
Backscattering suppression and *Q*_0_ in a high-Q resonator. **a** Backscattering amplitude measurement grid, with the resonator boundary and *critical tip coupling* line annotated. **b** Radial dependence of the backscattering suppression (circles, measurement; line, fit) along a radial line. The noise of the photodetector is indicated by the dotted grey line. Inset: suppression for all positions. The *critical tip coupling* at which the maximum suppression occurs is annotated. The measured suppression is limited by the photodetector noise. **c** Radial dependence of resonator and tip quality factor *Q*_0_ along a radial line. Inset: *Q*_0_ for all positions

Figure 4 shows data from a similar measurement for a resonator with *Q* $\simeq$ 1.1 × 10^9^. Due to the extremely high Q-factor, this measurement was performed with a lower scanning speed (10 GHz s^−1^) to avoid ring-down signals, calling for a lower input power (∼40 µW) to avoid thermal broadening. The backscattering pattern now shows a decrease in the backscattering for all azimuthal positions because the backscattering is linewidth dependent (see “Materials and methods” section), and the linewidth decreases more than for the resonator in Fig. 3. The maximum backscattering suppression obtained, 34 dB, is limited by the photodetector noise.

The total Q-factor of the system was calculated from $Q = \omega _{{\mathrm{pump}}}/(2\gamma )$ for pump frequency *ω*_pump_ = 2*π* × 193.1 GHz. Given the effective taper coupling *η*, the quality factor *Q*_0_ of the resonator with the tip present can be estimated as $Q_0 = 2Q/(1 + \sqrt {1 - \eta } )$, shown for different radial positions of the tip in Fig. 4c. The maximum suppression occurs when *Q*_0_ = 2.5 × 10^8^.

## Discussion

Optical microresonators provide prospects for miniaturised sensing and communications systems; however, backscattering compromises the performance of some microresonator-based systems. We have demonstrated a method for coherently suppressing the intrinsic backscattering in an optical WGM microresonator, with the suppression exceeding 34 dB (limited by photodetectors) from a level where frequency splitting is not resolved. Suppression of backscattering opens opportunities for pure travelling-wave resonators, improving the performance in microresonator applications where backscattering is a limiting factor. These applications include symmetry-breaking-based sensing or non-reciprocal systems, optomechanics applications, laser gyroscopes and dual frequency combs; thus, backscattering suppression enables the development of high-accuracy, portable optical spectroscopy systems, gyroscopes and other sensors. The technique is of particular interest for on-chip resonators, where a scatterer can be permanently integrated on chip to coherently suppress back reflections. In addition, one could envision tuneable on-chip backscattering suppression with a MEMS-based device.

## Materials and methods

### Resonator and tapered fibre fabrication

The rod resonators were machined using a 100 W CO_2_ laser, milling commercially available 3-mm-diameter silica glass rods. The procedure followed that of Del’Haye et al.^44^, but to reach the highest Q-factor of 10^9^, the resonator was fabricated in a nitrogen atmosphere, seeking to avoid the formation of near-IR-absorbing OH groups. The rod was fixed to a spindle motor, and the glass was evaporated with the focused laser beam. First, the low (high) Q rod was milled down to a 2.7-mm-diameter (1.7-mm-diameter) cylindrical shape, and then, the resonator was created by making two ring cuts separated by ∼125 µm (∼240 µm).

The tapered fibre was made from a stripped 125-µm-diameter standard single-mode silica optical fibre. The fibre was clamped to stepper motor stages, with a hydrogen flame placed under the fibre to heat it while simultaneously pulling symmetrically from both sides.

### Tungsten tip fabrication

The fabrication of the tungsten tip was based on methods used in scanning tunnelling electron microscopy and atomic force microscopy tip fabrication^45,46^. The process relies on surface tension to form a meniscus of a solution around a piece of tungsten wire and on the aqueous electrochemical reaction

$${\mathrm{W}}\left( {{\mathrm{solid}}} \right) + 2{\mathrm{OH}}^ - + 2{\mathrm{H}}_{\mathrm{2}}{\mathrm{O}} \to {\mathrm{WO}}_4^{2 - } + 3{\mathrm{H}}_{\mathrm{2}}\left( {{\mathrm{gas}}} \right)$$

etching the solid tungsten (W) anode through oxidation. When the minimum potential difference (1.43 V) is overcome, the etching rate varies along the wire due to a hydroxide concentration gradient: the etching rate is slower at the top of the meniscus because the ‘hydroxide supply’ is lower in the meniscus above the horizontal surface. This causes a tip shape to form. Further down, the wire is protected as the tungstate (WO_4_^2−^) ions fall along the sides of the wire, forming an increasingly dense laminar layer that protects the lower end of the tip from being etched. When the diameter at the meniscus is sufficiently decreased, gravity will break off the lower part of the wire.

Temper-annealed, 250-µm-diameter, 99.95% purity polycrystalline tungsten wire was used in the fabrication. The electrolyte was made by dissolving potassium hydroxide (KOH) in deionised water, producing a 7.5 mol L^−1^ concentration aqueous solution. The second electrode used was a tinned copper electrical wire with a diameter of 0.3 mm. The tungsten wire was pre-etched for five seconds at 4 V to reduce the surface roughness. After pre-etching, the wire was lifted ∼1 mm before continuing the etching process at 4 V until the lower part fell off. The total etching time was approximately two minutes.

### Experimental setup

A fibre-coupled external cavity diode laser at 1.55 µm, connected to an erbium-doped fibre amplifier, was used as the light source in the experiments. To obtain spectral measurements of the microresonator mode, the frequency of the laser source was scanned by current modulation with a triangular wave signal at 1007 Hz (Fig. 3) and 20 Hz (Fig. 4). As the light was subsequently fed into an amplifier operating at saturation, the optical power was kept constant. A polarisation controller was used for optimising the coupling to a resonator mode, and a circulator was used to separate the backward-propagating light in the tapered fibre for detection. The tapered fibre was mounted on a manual piezo stage to control the coupling to the resonator. Amplified photodetectors and an oscilloscope were used for simultaneously monitoring the transmitted and backscattered light.

The tungsten tip was fixed to a polylactic acid (PLA) plastic mount sitting on a three-axis piezo positioner. The tip position was raster scanned over a 1 µm × 1.5 µm area in the resonator plane, and backscattering and transmission spectra were obtained for each 50 nm (Fig. 3) or 25 nm (Fig. 4) step. The tip positioner and oscilloscope were simultaneously computer controlled, allowing a capture time of a few minutes for each of the two measurements reported.

### Data analysis

Least squares fitting procedures were applied to the spectra obtained in the measurement to determine the pump transmission half-linewidth *γ* and backscattering amplitude *A*_b_ of the cavity for each position in the measurement. No spectrally resolvable mode splitting was observed.

For the pump resonance, a normal Lorentzian dip from a background *B* was used, $B - {A_{\mathrm{p}}}/(1 + \delta ^2/\gamma ^2)$, where the detuning with respect to the resonance angular frequency *ω*_0_ is *δ* = *ω−ω*_0_. However, the spectral shape for the backscattering is distorted, as it is effectively pumped by a Lorentzian pulse (the pump resonance), resulting in a lineshape of *A*_b_/(1 + *δ*^2^/*γ*^2^)^2^_._ in the small backscattering limit (see Supplementary information for the derivation).

Subsequent to fitting the individual measurements, the grid data of the linewidth and backscattering amplitude measurements were fitted. The functions used for the grid data fitting are expressed in a rotated (Cartesian) coordinate system (*r, ϕ*) at an angle *β* to the measurement coordinate system (*x, y*), where the coordinate transformation is given by

$$\left( {\begin{array}{*{20}{c}} r \\ \phi \end{array}} \right) = \left( {\begin{array}{*{20}{c}} {\cos \beta } & { - \sin \beta } \\ {\sin \beta } & {\cos \beta } \end{array}} \right)\left( {\begin{array}{*{20}{c}} x \\ y \end{array}} \right)$$

In this coordinate system, the *r* axis is normal to the resonator surface and can be approximated as the azimuthal position over a short distance compared to the resonator radius of curvature. The rotation angle *β* was determined as one of the free parameters of the linewidth grid fit, where the linewidth function expressed in the (*r, ϕ*) coordinate system is

$$\gamma \left( {r,\phi } \right) = \gamma _0 + \left\{ {\begin{array}{*{20}{l}} {a_{\rm{p}} + s\left( {r - r_0} \right)} \\ {a_{\rm{p}}e^{ - 2\alpha _\gamma (r - r_0)}} \end{array}} \right.\begin{array}{*{20}{l}} {{\mathrm{for}}\;r - r_0 \, < \, 0} \\ {{\mathrm{for}}\;r - r_0 \ge 0} \end{array}$$

with the unperturbed linewidth *γ*_0_, decay coefficient *α*_*γ*_, amplitude of the exponential decay *a*_p_, linear slope of the plateau at the resonator surface *s*, and coordinate system offset *r*_0_ as free parameters.

The relative backscattering amplitude grid was subsequently fitted with the parameters *β* and *r*_0_ fixed to the values obtained from the linewidth fit. Only the portion of data outside the resonator boundary, $r - r_0 = R \ge 0$, was fitted. The function fitted for the backscattering amplitude *A*_b_ is derived in the Supplementary information and reads

$${A_{\rm{b}}} = \left\{ {\begin{array}{*{20}{l}} {\rm{not}}\,{\rm{fitted}}&{{\rm{for}}\, R \, < \,0} \\ {{{\vert g \vert}^{2}}/{{\gamma}^{4}}(r,\phi )}&{{\rm{for}}\,R\, \ge \,0} \end{array}} \right.$$

where *γ*(*r, ϕ*) is the fitted linewidth function, and the coupling from the forward-propagating to the back-propagating mode is

$$\left| g \right|^{2} = g_{0}^{2} + 2g_{0}a_{\mathrm{t}}e^{-2{\alpha}_{\mathrm{b}}R}{\cos} \left( {\Theta} \right) + a_{\mathrm{t}}^{2}e^{- 4{\alpha}_{\mathrm{b}}R}$$

where *γ*(*r, ϕ*) is the fitted linewidth function, *g*_0_ is the intrinsic backscattering strength, and ${\mathrm{{\Theta}}} = k_{{\mathrm{fr}}}\phi + \theta + \theta _RR$ is a position-dependent phase responsible for the fringe pattern, in which *θ*_*R*_ is a radially dependent phase accounting for the shape of the tip and/or drift. The period ∆ of the fringe pattern is given by $k_{{\mathrm{fr}}} = 2\pi /{\mathrm{{\Delta}}}$.

To sample arbitrary lines in the two-dimensional grid of measurement data shown in Figs. 3c–e and 4b, c, the grid data were linearly interpolated.

## Supplementary information

Supplementary Information

## Data Availability

The data that support this article are available from the corresponding author upon reasonable request.

## Code availability

The code for data analysis that supports the plots within this article is available from the corresponding author upon reasonable request.
