# Supplementary material for: Coherent suppression of backscattering in optical microresonators
Source: Light Sci Appl. 2020 Dec 23;9:204. doi: 10.1038/s41377-020-00440-2 (PMC7755905; doi:10.1038/s41377-020-00440-2)
Supplement: Supplementary file 1 — Supplementary Information [file 41377_2020_440_MOESM1_ESM.pdf]

Supplementary information: Coherent suppression of backscattering in optical microresonators

Andreas Ø. Svela,[1,2,3,*] Jonathan M. Silver,[1,4] Leonardo Del Bino,[1,5,6]
Shuangyou Zhang,[1,5] Michael T. M. Woodley,[1,6]
Michael R. Vanner,[2,3] and Pascal Del'Haye[1,5,7,†]

[1] *National Physical Laboratory, Teddington, TW11 0LW, UK*
[2] *Blackett Laboratory, Imperial College London, SW7 2AZ, UK*
[3] *Clarendon Laboratory, University of Oxford, OX1 3PU, UK*
[4] *City, University of London, EC1V 0HB, UK*
[5] *Max Planck Institute for the Science of Light, Staudtstaße 2, 91058 Erlangen, Germany*
[6] *Heriot-Watt University, Edinburgh, Scotland, EH14 4AS, UK*
[7] *Friedrich Alexander University Erlangen-Nuremberg, 91058 Erlangen, Germany*

The pump and backscatter lineshapes. Following a time-dependent approach[1–3], the steady-state equations of motion for the two circulating, counter-propagating fields $e_{\mathrm{cw,ccw}}$, both detuned by δ from the pump and perturbed by complex scattering coefficients g_{jk}, can be expressed as

$$\begin{pmatrix}\dot{e}_{\mathrm{cw}}\\ \dot{e}_{\mathrm{ccw}}\end{pmatrix} = \begin{pmatrix}-\gamma - i\delta + ig_{11} & ig_{12}\\ ig_{21} & -\gamma - i\delta + ig_{22}\end{pmatrix}\begin{pmatrix}e_{\mathrm{cw}}\\ e_{\mathrm{ccw}}\end{pmatrix} + \begin{pmatrix}E_{\mathrm{cw}}\\ 0\end{pmatrix} = 0$$

when a field E_{cw} is pumping the clockwise-propagating mode. Inverting the matrix, we obtain

$$\begin{pmatrix}e_{\mathrm{cw}}\\ e_{\mathrm{ccw}}\end{pmatrix} = \frac{E_{\mathrm{cw}}}{(\gamma + i\delta - ig_{11})(\gamma + i\delta - ig_{22}) + g_{12}g_{21}}\begin{pmatrix}\gamma + i\delta - ig_{22}\\ ig_{21}\end{pmatrix}.$$

In the small-backscattering regime $|g_{jk}| \ll \gamma$, this gives

$$e_{\mathrm{cw}} = \frac{E_{\mathrm{cw}}}{\gamma + i\delta}; \qquad e_{\mathrm{ccw}} = \frac{ig_{21}E_{\mathrm{cw}}}{(\gamma + i\delta)^2}, \tag{S1}$$

where $|e_{\mathrm{cw}}|^2, |e_{\mathrm{ccw}}|^2$ are proportional to the powers circulating in the respective directions.

The output fields in the taper from the cw and ccw directions can be expressed[1] using the taper coupling linewidth κ,

$$E_{\mathrm{cw,\,trans}} = E_{\mathrm{cw}} - 2\kappa e_{\mathrm{cw}}; \qquad E_{\mathrm{ccw,\,trans}} = E_{\mathrm{ccw}} - 2\kappa e_{\mathrm{ccw}} = -2\kappa e_{\mathrm{ccw}}.$$

To find the lineshapes of the output fields, insert Eq. (S1) and take the modulus squared to obtain

$$|E_{\mathrm{cw,\,trans}}|^2 = |E_{\mathrm{cw}}|^2\left(1 - \frac{4\kappa(\gamma-\kappa)}{\gamma^2+\delta^2}\right); \qquad |E_{\mathrm{ccw,\,trans}}|^2 = |g_{21}|^2|E_{\mathrm{cw}}|^2\frac{4\kappa^2}{(\gamma^2+\delta^2)^2}.$$

The total linewidth has two components, the intrinsic losses γ_0 and the taper coupling κ, such that $\gamma = \gamma_0 + \kappa$. Furthermore, the coupling efficiency $\eta = 4\kappa\gamma_0/\gamma^2$, giving

$$|E_{\mathrm{cw,\,trans}}|^2 = |E_{\mathrm{cw}}|^2\left(1 - \frac{\eta}{1+\delta^2/\gamma^2}\right); \quad |E_{\mathrm{ccw,\,trans}}|^2 = |g_{21}|^2|E_{\mathrm{cw}}|^2\frac{4\kappa^2/\gamma^4}{(1+\delta^2/\gamma^2)^2}. \tag{S2}$$

This shows that the transmitted clockwise pump has a dip with a normal Lorentzian lineshape, whereas the backscattered power will exhibit a peak with a squared Lorentzian lineshape of amplitude $A_{\mathrm{b}} \propto |g_{21}|^2/\gamma^4$.

* asvela@ic.ac.uk
† pascal.delhaye@mpl.mpg.de

Derivation of the backscattering fitting function. From Eq. (S2), we are expecting the backscattering power at resonance to be proportional to $|g_{21}|^2/\gamma^4$. The coupling coefficient g_{21} has two contributions, an intrinsic g_0, which by a suitable choice of the relative phase between the cw and ccw basis states can be made to be real, and a tip-induced $g_{\rm tip}$. The tip-induced coupling is expected to follow $g_{\rm tip} = a_{\rm t}\, e^{-2\alpha_{\rm b}R}e^{i\Theta}$, where $\Theta = k_{\rm fr}\phi + \theta + \theta_R R$ is a position-dependent phase responsible for the fringe pattern, in which θ_R is a radially dependent phase accounting for the shape of the tip and/or drift. Coherently adding the two contributions, $|g_0 + g_{\rm tip}|^2$, we obtain

$$|g_{21}|^2 = g_0^2 + 2g_0 a_{\rm t}\, e^{-2\alpha_{\rm b}R}\cos(\Theta) + a_{\rm t}^2\, e^{-4\alpha_{\rm b}R}.$$

As only the portion of data outside the resonator boundary, $r - r_0 = R \geq 0$, was fitted, the fitting function for the backscattering amplitude can be expressed as

$$A_{\rm b}(R,\phi) = \begin{cases} \text{not fitted} & \text{for } R < 0 \\ \dfrac{g_0^2 + 2g_0 a_{\rm t}\, e^{-2\alpha_{\rm b}R}\cos\left[\Theta(R,\phi)\right] + a_{\rm t}^2\, e^{-4\alpha_{\rm b}R}}{\gamma^4(r,\phi)} & \text{for } R \geq 0 \end{cases}, \tag{S3}$$

in which $\gamma(r,\phi)$ is the fitted linewidth function.

Expected period of the fringe pattern. The fringe pattern in the backscattering arises from the $\cos(\Theta)$ term in Eq. (S3), where $\Theta(R,\phi)$ is the phase of the backscattered light from the tip. We find the periodicity Δ of the fringe pattern with respect to the azimuthal position ϕ by considering the distance between subsequent maxima. As the phase of the backscattered light depends on the phase of the clockwise mode relative to the ccw mode at the position of each part of the tip involved in backscattering, this relative phase difference varies with ϕ by $2k_{\rm opt}\phi$, where $k_{\rm opt}$ is the optical wavenumber. Hence, the distance Δ between maxima is given by $\Theta_{m+1} - \Theta_m = 2\pi = 2k_{\rm opt}(\phi_{m+1} - \phi_m) = 2k_{\rm opt}\Delta$.

For a resonator of material with refractive index n, the optical wavenumber $k_{\rm opt} \simeq 2\pi n/\lambda$ for the vacuum wavelength λ (the approximation is due to the transverse confinement of the mode), giving a period $\Delta \simeq \lambda/(2n)$.

---

[1] Rabus, D. G. *Integrated Ring Resonators*, vol. 127 of *Optical Sciences* (Springer, 2007).

[2] Wiersig, J. Structure of whispering-gallery modes in optical microdisks perturbed by nanoparticles. *Phys. Rev. A* **84**, 063828 (2011).

[3] Woodley, M. T. M. *et al.* Universal symmetry-breaking dynamics for the Kerr interaction of counterpropagating light in dielectric ring resonators. *Phys. Rev. A* **98**, 053863 (2018).
